# Mint companion plants attract the predatory mite *Phytoseiulus persimilis*

**DOI:** 10.1038/s41598-018-38098-x

**Published:** 2019-02-08

**Authors:** Kazuki Togashi, Mifumi Goto, Hojun Rim, Sayaka Hattori, Rika Ozawa, Gen-ichiro Arimura

**Affiliations:** 1 Department of Biological Science & Technology, Faculty of Industrial Science & Technology, Tokyo University of Science, Tokyo, 125-8585 Japan; 2 Center for Ecological Research, Kyoto University, Otsu, 520-2113 Japan

## Abstract

Mint plants could theoretically serve as companion plants (CPs) that attract enemies of herbivores in tritrophic interactions. In order to explore the traits of mint volatiles as attractant cues for enemies of two-spotted spider mites, we performed Y-tube olfactometer assays of predatory mites, *Phytoseiulus persimilis* and *Neoseiulus californicus*, towards three mint species, apple mint, candy mint, and spearmint, as odor source. Clean candy mint and spearmint were attractive to *P. persimilis*, when compared with clean air and undamaged *Phaseolus vulgaris* plants serving as the target crop. Moreover, clean candy mint plants were even more attractive than volatiles from *P. vulgaris* plants damaged by spider mites. These predator responses were induced additively by candy mint volatiles plus volatiles from damaged *P. vulgaris* plants, as shown using both Y-tube olfactometer and open-space assay systems. However, the number of spider mite eggs consumed by *P. persimilis* on *P. vulgaris* plants did not differ in the presence compared to the absence of mint volatiles, indicating that mint volatiles affect the attraction but not the appetite of *P. persimilis*. Together, these findings suggest that the use of candy mint and spearmint as CPs is an ideal platform for spider mite pest management via the attraction of predatory mites.

## Introduction

Volatile organic compounds (VOCs) are released into the atmosphere for many purposes from plants belonging to a vast array of taxa to play important roles in attracting mutualistic animals, resisting environmental stress, and directly controlling plant pests^1,2^. One class of plant-produced VOCs is released to promote tritrophic interactions among the plant, its herbivores, and herbivore enemies (predators and parasitoids), implying that VOCs contribute to plant indirect defense responses by attracting plants’ bodyguards^2^.

In light of this, the nature of plant indirect defenses that act in the tritrophic interactions among legume plants, herbivorous two-spotted spider mites (*Tetranychus urticae*), and predatory mites (e.g., *Phytoseiulus persimilis* and *Neoseiulus californicus*) has been intensively studied^3–6^. *T. urticae* is an agricultural pest of a broad range of herbaceous and woody plants throughout the world^3,7^, and this pest is able to quickly evolve resistance to acaricides due to its short life span and relatively high fecundity, and arrhenotokous reproduction^8,9^. Therefore, studies targeting plant indirect defenses provide an appropriate basis for the development of new protocols for *T. urticae* pest management that do not rely on acaricides in agriculture and horticulture.

“Companion planting” is one specific type of polyculture in which target plants (TPs) are cultivated with companion plants (CPs) to assist TP growth or protection against pests by attracting beneficial insects or repelling pests^10^. Various applications of CPs have been developed for pest management, e.g., disrupting the ability of herbivores to locate TPs by directly repelling herbivores or by masking host odors via VOCs released from CPs^11^, or trapping of herbivore pests by using CPs^12^. Moreover, CPs such as mint, basil and marigold work as attractants for herbivore enemies^13,14^. For example, coriander plants have been shown to attract the ladybug *Cycloneda sanguinea*, an aphid predator, and the predator attracted onto coriander plants uses coriander pollen and nectar as supplementary foods when aphids are not available^15^.

Regarding mints (*Mentha* spp.) that serve as CPs, aromatic essential oils (including VOCs) of *M. pulegium* L.*, M. longifolia* L. and *M. × piperita* L. have been shown to exhibit toxicity against eggs of *T. urticae* and repellent activity towards *T. urticae* adults^16–18^. However, since little is known about the attractivity of mint volatiles for herbivore enemies, we assessed the attraction of two predatory mites (*P. persimilis* and *N. californicus*) to mint VOCs using a Y-tube olfactometer. As odour sources, we tested candy mint (*M*. × *piperita* L. cv. Candy), spearmint (*M. spicata* L.), and apple mint (*M. suaveolens*), which have been categorized into three distinct types, namely, cool-pungent (candy mint), cool-sweet (spearmint), and cool-fruity (apple mint)^19^. Given the significant role shown here of mint volatiles in attracting *P. persimilis*, we suggest a potential application of these mint species for *T. urticae* pest management.

## Results

### Olfactory responses of the predatory mites to mint VOCs

**Figure 1:**
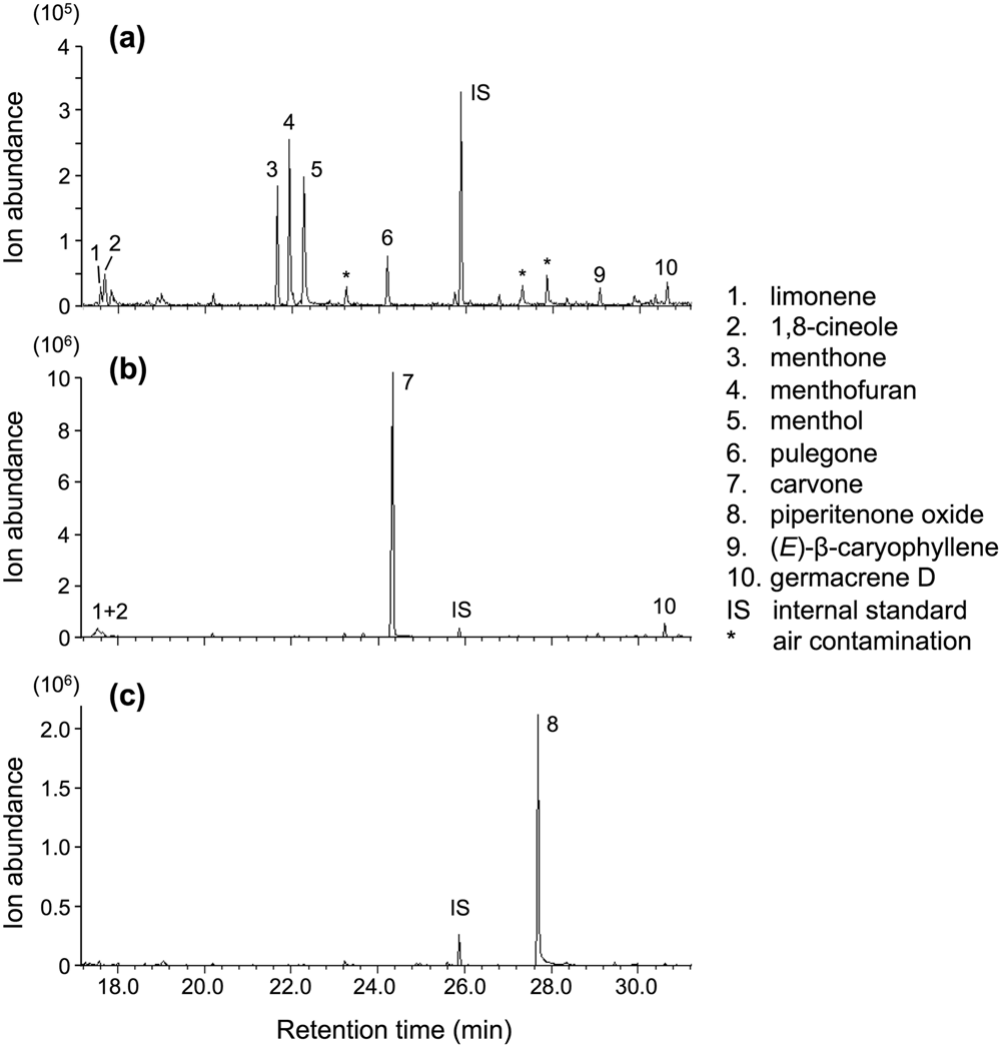
Headspace VOC profiles of the plantlets of candy mint (**a**), spearmint (**b**), and apple mint (**c**).

As shown in Fig. 1, plantlets of candy mint (*M*. × *piperita* cv. Candy), spearmint (*M. spicata* L.), and apple mint (*M. suaveolens*) emitted VOCs with distinct profiles. The VOCs emitted from candy mint consisted of monoterpenes, including 1,8-cineole, menthone, menthofuran, menthol, and pulegone. In contrast, spearmint and apple mint predominantly released only a few major monoterpene components, carvone and piperitenone oxide.

**Figure 2:**
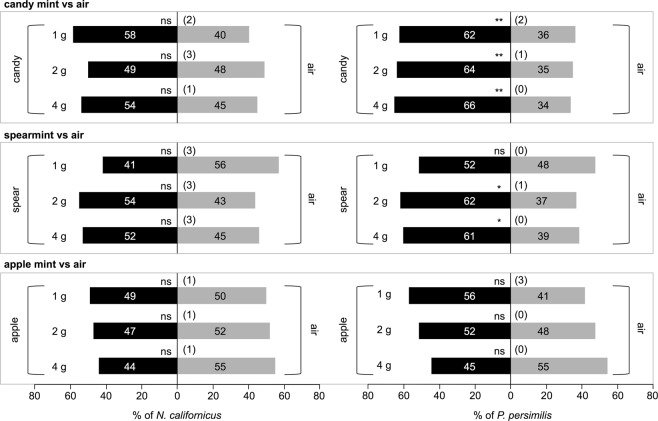
Olfactory response of *Neoseiulus californicus* or *Phytoseiulus persimilis* when offered three species of mint plantlets (1, 2 or 4 grams fresh weight) vs clean air in a Y-tube olfactometer. The numbers in the bars indicate the numbers of predatory mite females that made choices. The figures in parentheses represent the numbers of predators that did not choose either odor source (‘no choice’ subjects). A replicated G-test was conducted to evaluate the significance of attraction in each experiment (**0.001 ≤ *P* < 0.01; *0.01 ≤ *P* < 0.05; ns, *P* > 0.05).

We assessed the effects of those VOCs on the olfactory responses of the predatory mites *P. persimilis* and *N. californicus* when compared with clean air, using a Y-tube olfactometer. *N. californicus* did not show any preference for the VOCs emitted from any of the mint plantlets of 1, 2 or 4 grams fresh weight (gFW) (*P* > 0.05; Fig. 2). In contrast, *P. persimilis* was significantly attracted to candy mint (1, 2 and 4 gFW; *P* < 0.01) and spearmint (2 and 4 gFW; *P* < 0.05) but not apple mint (1, 2 and 4 gFW; *P* > 0.05). Based on these results, we concluded that candy mint and spearmint volatiles serve as attractants for *P. persimilis*, and used them for subsequent assays.

**Figure 3:**
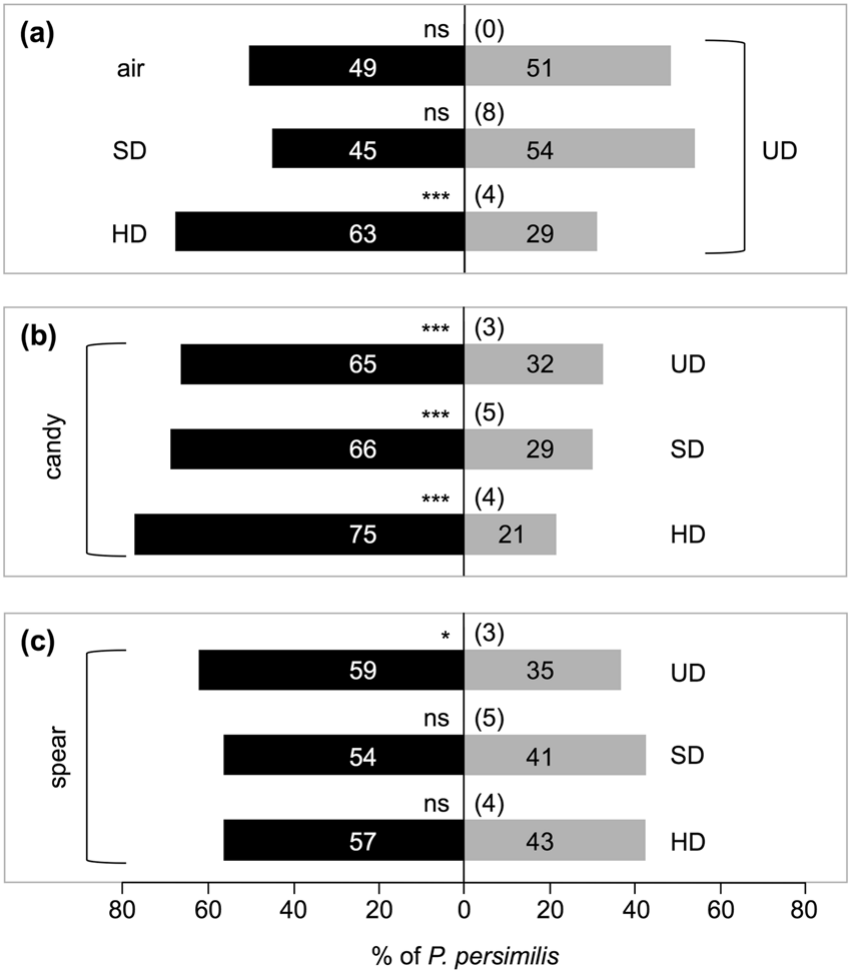
Olfactory response of *Phytoseiulus persimilis* when offered clean air, or potted, slightly damaged (SD) *P. vulgaris* plants or heavily damaged (HD) *P. vulgaris* plants vs. potted, undamaged (UD) Phaseolus vulgaris plants (**a**) and when offered candy mint or spearmint plantlets (4 g fresh weight) vs. UD, SD or HD plants (**b**,**c**) in a Y-tube olfactometer. The numbers in the bars indicate the numbers of predatory mite females that made choices. The figures in parentheses represent the numbers of predators that did not choose either odor source (‘no choice’ subjects). A replicated G-test was conducted to evaluate the significance of attraction in each experiment (***P < 0.001; *0.01 ≤ *P* < 0.05; ns, *P* > 0.05).

We then observed that candy mint plantlets (4 gFW) were more attractive for *P. persimilis* than potted, undamaged (UD) *P. vulgaris* plants or *P. vulgaris* plants damaged slightly or heavily with *T. urticae* (hereafter referred to as SD and HD, respectively) (Fig. 3b). Spearmint (4 gFW) was more attractive than UD plants but not SD or HD plants (Fig. 3c). Since HD plants were more attractive than SD and UD plants (Fig. 3a), we concluded that the relative attractiveness was: candy mint > HD  = spearmint > UD.

### Identification of attractive mint VOCs

To identify the attractive components among the VOCs emitted from candy mint (limonene, 1,8-cineole, menthone, menthofuran, menthol, pulegone, and (*E*)-β-caryophyllene; Fig. 1), authentic VOCs released from hexane solutions were assessed for the olfactory responses they induced in the predatory mite *P. persimilis*. We assessed the effects of levels of those VOCs that approximately corresponded to the headspace levels of major VOCs released from 4 gFW of candy mint^20^, and found that none of the components alone were attractive to the predatory mites, when compared with clean air (*P* > 0.05; Supplemental Fig. 1). Next, we assessed a blend of VOCs in which the level of each of the VOC components corresponded approximately to its headspace level in candy mint VOCs, and we again observed no preference for the blend over clean air (*P* > 0.05). The same held true for carvone at levels that approximately corresponded to its headspace level in VOCs released from 4 gFW of spearmint as well as a blend of VOC components consisting of carvone, limonene and 1,8-cineole.

### Additive effect of mint VOCs with *T. urticae*-induced plant volatiles

**Figure 4:**
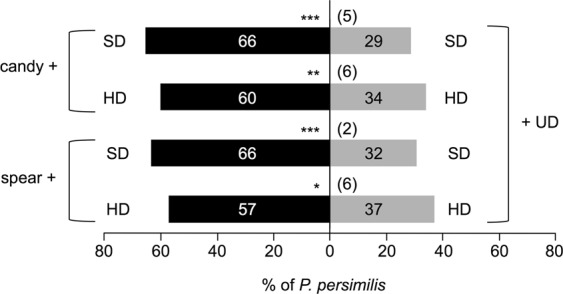
Olfactory response of *Phytoseiulus persimilis* when offered candy mint or spearmint plantlets (4 g fresh weight) + potted, slightly damaged (SD) *Phaseolus vulgaris* plants or heavily damaged (HD) *P. vulgaris* plants vs. SD plants + potted, undamaged (UD) *P. vulgaris* plants or HD plants + UD plants in a Y-tube olfactometer. The numbers in the bars indicate the numbers of predatory mite females that made choices. The figures in parentheses represent the numbers of predators that did not choose either odor source (‘no choice’ subjects). A replicated G-test was conducted to evaluate the significance of attraction in each experiment (***P < 0.001; **0.001 ≤* P* < 0.01; *0.01 ≤ *P* < 0.05).

To deepen our understanding of the attractivity of mint VOCs, we next assessed the attractivity of mint plantlet (4 gFW) VOCs blended with VOCs from potted SD or HD plants (Fig. 4). *P. persimilis* preferred candy mint or spearmint VOCs blended with SD or HD plant volatiles over VOCs from either SD or HD plant volatiles blended with UD plant volatiles serving as background control (*P* < 0.05). These results demonstrated that mint VOCs work additively with *T. urticae*-induced plant volatiles for predator attraction. Moreover, we considered the possibility that mint VOCs may influence the ability of the damaged neighboring plants to emit VOCs, a phenomenon frequently referred to as “eavesdropping”^20^. To test this possibility, we used an assay system in which mint plantlets and a potted *P. vulgaris* plant were separately set in independent containers and then the odor cues from the two containers were mixed in front of an arm of a Y-tube olfactometer (see Supplemental Fig. 2a). We found that the attractivity of the mixed VOCs from these two containers containing the mint plantlets and HD plant separately was not different from that from a single container containing both together (*P* > 0.05; Supplemental Fig. 2b), thus ruling out an eavesdropping effect.

### Attractivity of mint VOCs in an open space

**Figure 5:**
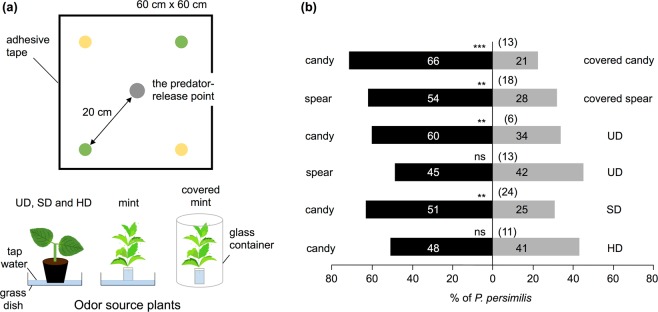
Behavior of *Phytoseiulus persimilis* towards mint plants in an open space. (**a**) Schematic drawing of experimental setup for the assay. (**b**) A pair of candy mint or spearmint plantlets was placed at diagonal positions at the corners, and another pair of odor-source plants was placed at the other corners, on a square board at the same distance from the predator-release point. The odor sources used were: mint plantlets; covered mint plantlets; and potted, undamaged (UD), slightly damaged (SD), or highly damaged (HD) *Phaseolus vulgaris* plants. Bars represent the overall percentages of predatory mites choosing either of the odor sources. The numbers in the bars indicate the numbers of predatory mite females that made choices. The figures in parentheses represent the numbers of predators that did not choose either odor source (‘no choice’ subjects). A replicated G-test was conducted to evaluate the significance of attraction in each experiment (***P < 0.001; **0.001 ≤* P* < 0.01; ns, *P* > 0.05).

The plantlets of both candy mint and spearmint (4 gFW) when placed on a white board (60 cm × 60 cm) were preferred by *P. persimilis* as compared to the respective mint plantlets that were covered with a glass container on the board to interrupt airborne interaction via mint VOCs (*P* < 0.05; Fig. 5). This demonstrated that odorant cues rather than visible cues served for attraction of the predator. Likewise, candy mint was more strongly preferred compared to potted UD or SD plants (*P* < 0.05) but not compared to HD plants. In accord with the results of the Y-tube olfactometer assays, spearmint was not preferable to the UD plants.

### Predation activity in the presence of mint VOCs

**Figure 6:**
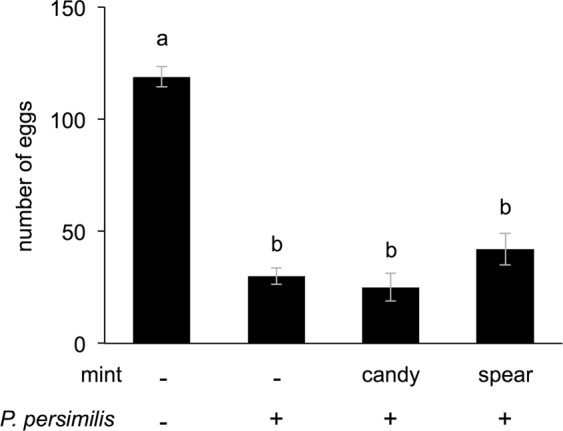
Predation ability of *Phytoseiulus persimilis*. Twenty adult female spider mites were placed on a potted *Phaseolus vulgaris* plant for 24 h, resulting in oviposition of approximately 120 eggs (Mint: −; *P. persimilis*: −). Two adult females of *P. persimilis* were placed on a potted *Phaseolus vulgaris* plant with nearby candy mint or spearmint plantlets (4 g fresh weight) or not (−), and the intact eggs were counted after 48 h. Data represent the means and standard errors (*n* = 4–5). The means indicated by different small letters are significantly different based on an ANOVA with post hoc Tukey’s HSD (*P* < 0.05).

Finally, we evaluated the total number of eggs consumed by *P. persimilis* on the potted *P. vulgaris* plants in the presence or absence of candy mint or spearmint plantlets (4 gFW) during 2 days (Fig. 6). The predator consumed about 75% of the eggs during 2 days. There was no significant difference between the consumption in the presence compared to the absence of mints (*P* > 0.05).

## Discussion

Our findings document that candy and spearmint may act as protective companion plants for neighboring bean plants in a tri-trophic context, by attracting the predator *P. persimilis* in the absence and presence of its prey *T. urticae*. Specifically, the mint VOCs attracted the predators but did not increase their appetite (Fig. 6). Our finding that candy mint was more attractive than uninfested or slightly *T. urticae*-infested bean plants in Y-tube olfactometer and open space assays indicates that candy mint has great potential as a companion plant (CP) for *T. urticae* pest management. Based on previous studies, several VOCs from *T. urticae*-infested plants, including (*E*)-β-ocimene, (*Z*)-3-hexen-1-ol, methyl salicylate, and (*E*,*E*)-4,8,12-trimethyltrideca-1,3,7,11-tetraene, have been proposed to contribute to the attraction of *P. persimilis*^4,6,21^, but none of these compounds were detected in VOCs from candy mint or spearmint (Fig. 1). To the best of our knowledge, no previous reports have shown that *P. persimilis* prefers aromatic compounds other than mite-induced plant volatiles. Moreover, our findings reveal the novel feature that the attraction of a predator may be based on not only its reward but also on a pre-existing sensory preference.

However, we still need to unravel the puzzling details of the function of mint VOCs. For instance, it remains to be elucidated why *P. persimilis* innately prefers mint VOCs. Its attraction to the VOCs from candy mint plants was even stronger than its attraction to VOCs from *T. urticae*-infested bean plants (Fig. 3). Curiously, we found that none of the authentic VOCs corresponding to the major mint VOCs are by themselves responsible for significant attraction of *P. persimilis* (Supplemental Fig. 1). Moreover, the tested blends of major mint volatiles were also not responsible for the attraction of *P. persimilis*. Here, we should point out that it is technically difficult to formulate the precise blend of plant volatiles that should be reconstructed by blending authentic VOCs. We infer that none of the single VOCs alone is responsible for the attraction of *P. persimilis*, and instead the precisely composed, natural blend of mint VOCs appears to be required for the attraction.

Moreover, it should be noted that evolutionary and ecological insights into the predators’s stronger attraction to mint plants than *T. urticae*-infested host plants remain to be obtained, but addressing those questions was beyond the scope of our study. There are some reports regarding similar phenomena of carnivores being attracted by VOCs that do not emanate from herbivore-damaged plants but rather from other plants, for instance, the reported attraction of *Harmonia axyridis* to French marigold^22^, *Ceraeochrysa cubana* to basil^23^, and ladybug to coriander plants^15^. However, whether their attraction is even stronger than that to herbivore-infested plants, and the ecological and evolutionary significance of their attraction, remain unknown. Although the relevance of background odor during rearing to subsequent resource location is of possible concern^24^, we tried to avoid this possibility during rearing of the predatory mites (see Materials and Methods).

Curiously, *N. californicus* was not responsive to any of the mint species (Fig. 2). This was unexpected because *P. persimilis* is a voracious, specialized predator of *Tetranychus* mites, whereas *N. californicus* is a generalized predator that consumes not only mites but also pollen, thrips, and other tiny arthropods^25^. Therefore, initially we expected that the generalist *N. californicus* rather than the specialist *P. persimilis* would be responsive to mint VOCs, whereas in fact we observed the opposite responses. For this reason, we suggest that perception of mint volatiles by *N. californicus* has not been selected for in an ecosystem containing mint host and *T. urticae*, unlike in the case of *P. persimilis*. Another possibility is that *N. californicus* may not prefer mint VOCs irrespective of its evolutionary experiences.

### Concluding remarks

The findings of our study provide new insights into potential application of selected mint cultivars for spider mite management in agriculture and horticulture. However, the exact mechanism that makes the predator move from mint to TP at the time when *T. urticae* starts to colonize the plants remains unknown. Visual effects produced by spider mites on TPs may help the migration of the predator, but it was reported that *P. persimilis* does not have a visual sensor^26^ and our results are in accord with this (Fig. 5). The preference of *P. persimilis* to stay in a prey patch rather than on a plant without its prey^27^ may support the immigration of the predator from CP to TP in the presence of prey mites.

In addition to attracting the predatory mites, candy mint VOCs appear to repel adult *T. urticae* females (Supplemental Fig. 3), a finding similar to the findings that the essential oils of Lamiaceae are repellent to the spider mites^16–18^. Together, our findings indicate that the mint VOCs should work synergistically for pest management, owing not only to their attractivity to predatory mites but also to their multi-functions in the ecosystem, for instance, repelling insect and mite herbivores and boosting anti-herbivore activity in neighboring crops^20^.

## Materials and Methods

### Plants

All of the plants used in the current study were incubated in climate-controlled rooms at 24 ± 1 °C with a photoperiod of 16 h (80 µE m^−2^ s^−1^). The light period was from 07:00 to 23:00. Apple mint (*M. suaveolens*), candy mint (*M*. × *piperita* cv. Candy), and spearmint (*M. spicata* L.) were obtained from gardening shops. The mint plants were transplanted to soil in plastic pots (8.5 cm diameter, 10.5 cm high) and cultivated in the above conditions. They were propagated by the stem-cutting method, and used when they had grown to sufficiently developed stages (at about 2 weeks). For use as odor sources, the mint plantlet(s) (approximately 1, 2, or 4 gFW) were cut and placed in a glass vial containing tap water (35 mL). Kidney bean *(Phaseolus vulgaris*, Fabaceae cv. Nagauzuramame) seeds were grown in plastic pots for 12 days. In order to avoid airborne contamination between mint and bean plants, all of the bean plants were cultivated at least 5 m away from mint plants when the plants were cultivated in the same room.

### Arthropods

*T. urticae* Koch (Acari: Tetranychidae) were reared on detached *P. vulgaris* leaf discs (25 cm^2^ each) placed on water-saturated cotton in Petri dishes (90 mm diameter, 14 mm depth) at 24 ± 1 °C. Small leaf discs (each 1 cm^2^), which were inhabited by about 20 mites and eggs, were collected from the original discs and transferred to fresh leaf discs every 2 weeks for incubation. Adult females (10 days old) after oviposition were used for assays.

*P. persimilis* (Phytoseiidae) and *N. californicus* (Phytoseiidae) were obtained from Arysta LifeScience (Tokyo, Japan) and reared continuously in the laboratory. They were placed on the leaf discs with spider mites as prey. New leaf discs with spider mites (see above) were provided to them every day. Fertilized female mites were used for the experiments at 5–10 days after their final moulting.

In order to avoid associative learning of the odor of mint volatiles by the predatory mites during rearing, all of the predatory mites were reared in seclusion from mint plants and with constant ventilation of the incubator room.

### Preparation of damaged plants

The potted bean plants were damaged with 20 or 100 spider mite adult females for 24 h, thus producing SD and HD plants, respectively.

### Preparation of authentic volatiles

The authentic compounds used were 1,8-cineole (Wako Pure Chemical Industrials, Ltd., Osaka, Japan), (*S*)-(−)-limonene (Wako), menthone (Wako), menthofuran (Extrasynthese, Genay, France), menthol (Wako), pulegone (Wako), and (*E*)-β-caryophyllene (Wako). An authentic chemical solution (0.19 mg mL^−1^ 1,8-cineole; 0.02 mg mL^−1^ (*S*)-(−)-limonene; 0.27 mg mL^−1^ menthone; 0.13 mg mL^−1^ menthofuran; 0.4 mg mL^−1^ menthol; 0.49 mg mL^−1^ pulegone; 0.15 mg mL^−1^ (*E*)-β-caryophyllene) in 3 mL of hexane in a glass vial (4 mL) was prepared to emit VOCs that approximately corresponded to the headspace levels of major VOCs released from candy mint (4 gFW)^20^. Similarly, an authentic chemical solution of (*S*)-(−)-limonene (see above), 1,8-cineole (see above), and (*R*)-(−)-carvone (1.6 mg mL^−1^, Wako) was prepared to emit VOCs that mimicked the headspace level of the VOCs from spearmint (4 gFW). A blend of authentic VOCs consisting of candy mint VOCs (1,8-cineole, (*S*)-(−)-limonene, menthone, menthofuran, menthol, pulegone, and (*E*)-β-caryophyllene) and spearmint VOCs ((*S*)-(−)-limonene [0.02 mg mL^−1^], 1,8-cineole [0.1 mg mL^−1^] and (*R*)-(−)-carvone) at the respective ratios corresponding to the headspace levels was also prepared in 3 mL of hexane in a glass vial. Note that germacrene D was excluded from our assays because it was not available as a pure authentic chemical.

### Y-tube olfactometer

Mint plantlet(s) in a glass vial, a potted *P. vulgaris* plant, or authentic VOCs in a glass vial (see above) or the corresponding control (hexane in a glass vial) was placed in a glass container (2 L) and used as the single-odor source. For assays whose results are shown in Supplemental Fig. 2, an HD plant and a candy mint or spearmint plant were enclosed in a single container or in two separate containers. We then assessed the olfactory responses of *P. persimilis*, *N. californicus* and *T. urticae* using a Y-tube olfactometer (3.5 cm inner diameter, 13 cm long for each branch tube and 13 cm long for the main tube). The adult female predators were starved overnight by placing 20 mites in a sealed plastic case containing wet cotton with water and used for assays, whereas non-starved *T. urticae* were used for assays. All of the herbivorous and predatory mites were individually introduced into the Y-shaped wire inside the olfactometer, and the numbers of mites choosing one of the odor sources were recorded. Mites that did not choose within 5 min (“no choice” subjects) were excluded from the statistical analysis. The orientation of the odor-source containers in the olfactometer arms was changed after every five bioassays. Assays using 20 mites were carried out as a single replicate in one day. Each assay was carried out on five different days (100 predators in all) with new sets of odor sources. The experiments were performed in a climate-controlled room (24 ± 1 °C).

### Predation assay

The assays were performed in a climate-controlled room (24 ± 1 °C) with a photoperiod of 16 h (80 µE m^−2^ s^−1^). Twenty adult female spider mites were incubated on a potted *P. vulgaris* plant for 24 h. After all the adult mites were removed, eggs that had been oviposited were counted to ensure the presence of approximately 100 eggs on the plant. Two adult females of the *P. persimilis* predator were then placed on the plant and reared with or without candy mint plantlets (4 gFW) for 48 h, and eggs that had not been eaten or damaged by the predators were counted. During the assays, plants were kept in a plastic box (26 × 34 cm, 34 cm high) with 2 mesh windows. Four or five replicates were performed.

### *P. persimilis* behavioral assay in an open space area

The assays were performed in a climate-controlled room (24 ± 1 °C) under light conditions. An adult female of the *P. persimilis* predator that had been starved overnight was released on the center of a white board of polyvinyl chloride (60 cm × 60 cm). A pair of odor-source plants were placed at diagonal corners, and another pair of odor-source plants was placed at the other corners, on the board at the same distance (20 cm) from the predator-release point (see Fig. 5a). The odor sources used were: 1) mint plantlets in a glass vial (4 gFW), 2) mint plantlets covered with a glass container (1.2 L, 10 cm diameter, 15.5 cm deep); 3) a potted UD *P. vulgaris* plant (approximately 4 gFW); 4) a potted SD *P. vulgaris* plant; 5) and a potted HD *P. vulgaris* plant. All of the plant samples were placed in a Petri dish (9 cm diameter). The predators that reached the Petri dish were immediately collected using a fine paintbrush and their numbers were recorded for 10 min after the onset of predator release. Predators that did not reach the dish within 10 min (“no choice” subjects) were excluded from the statistical analysis. Assays using 20 predators were carried out as a single replicate in a day. During the single replicate, the positioning of the odor-source plants was changed after every five behavioral assays. Each assay was carried out on five different days (100 predators in all) with new sets of odor sources, and the results were subjected to a replicated G-test.

### Headspace volatile analysis

Volatiles from mint plantlets in a glass vial (2 gFW) were collected in a glass container (2 L) using Tenax 60/80 (Gerstel GmbH & Co. KG, Mülheim an der Ruhr, Germany) in a laboratory room (24 ± 1 °C, under light conditions) for 1 h. Clean air passed through a charcoal filter was drawn into the glass container, and VOCs from the headspace of the container were collected at a flow rate of 100 mL min^−1^. *n*-Tridecane (0.1 μg) infiltrated into a piece of filter paper (1 cm^2^) was added to the glass container as an internal standard at the onset of VOC collection.

The collected volatile compounds were analyzed by gas chromatography-mass spectrometry (GC-MS) (GC: Agilent Technologies, Santa Clara, CA, USA; 6890 with an HP-5MS capillary column: 30 m long, 0.25 mm I.D., and 0.25 µm film thickness; MS: Agilent Technologies, a 5973 mass selective detector, 70 eV) equipped with a thermal desorption system, a cooled injection system, and a cold trap system (Gerstel GmbH & Co. KG). Headspace volatiles collected on a Tenax were released by heating in the thermal desorption system (TDS) at 280 °C for 4 min, within a He flow. Split injection of the TDS was conducted with a split ratio of 1:50. The desorbed compounds were collected in the cooled injection system (CIS) at −90 °C, and then the collected compounds were released from the CIS by heating (230 °C). The desorbed compounds were collected again in the cold trap system (CTS) at −50 °C, and then flash heating of the CTS (200 °C) provided sharp injection of the compounds into the capillary column of the gas chromatograph to which the CTS was connected. GC-oven temperature was programmed to rise from 40 °C (9 min hold) to 280 °C at 5 °C min^−1^. The headspace volatiles were identified by comparing their mass spectra and retention times with those of authentic compounds.

### Statistical analysis

A replicated G-test was conducted to evaluate the data from the Y-tube olfactometer analyses and open space assays. For predation assays, we performed one-way ANOVAs with Tukey’s HSD test using an online program (http://astatsa.com/OneWay_Anova_with_TukeyHSD/).

## Supplementary information

Supplementary Figures
