# Supplementary material for: Mint companion plants attract the predatory mite Phytoseiulus persimilis
Source: Sci Rep. 2019 Feb 8;9:1704. doi: 10.1038/s41598-018-38098-x (PMC6368615; doi:10.1038/s41598-018-38098-x)
Supplement: Supplementary file 1 — Supplementary Figures [file 41598_2018_38098_MOESM1_ESM.pdf]

Mint companion plants attract the predatory mite *Phytoseiulus persimilis*

Kazuki Togashi[1], Mifumi Goto[1], Hojun Rim[1], Sayaka Hattori[1], Rika Ozawa[2], Gen-ichiro Arimura[1]

[1]Department of Biological Science & Technology, Faculty of Industrial Science & Technology, Tokyo University of Science, Tokyo 125-8585, Japan
[2]Center for Ecological Research, Kyoto University, Otsu 520-2113, Japan

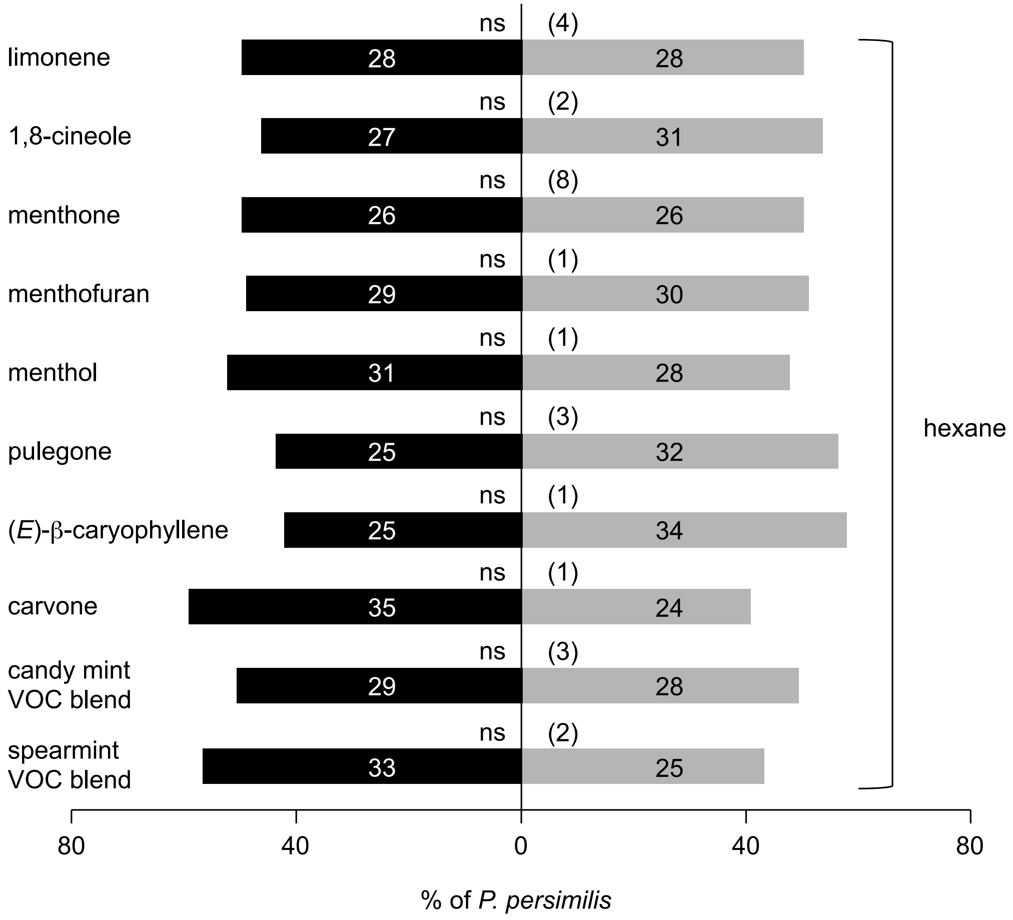

Supplemental Fig.1

(a)

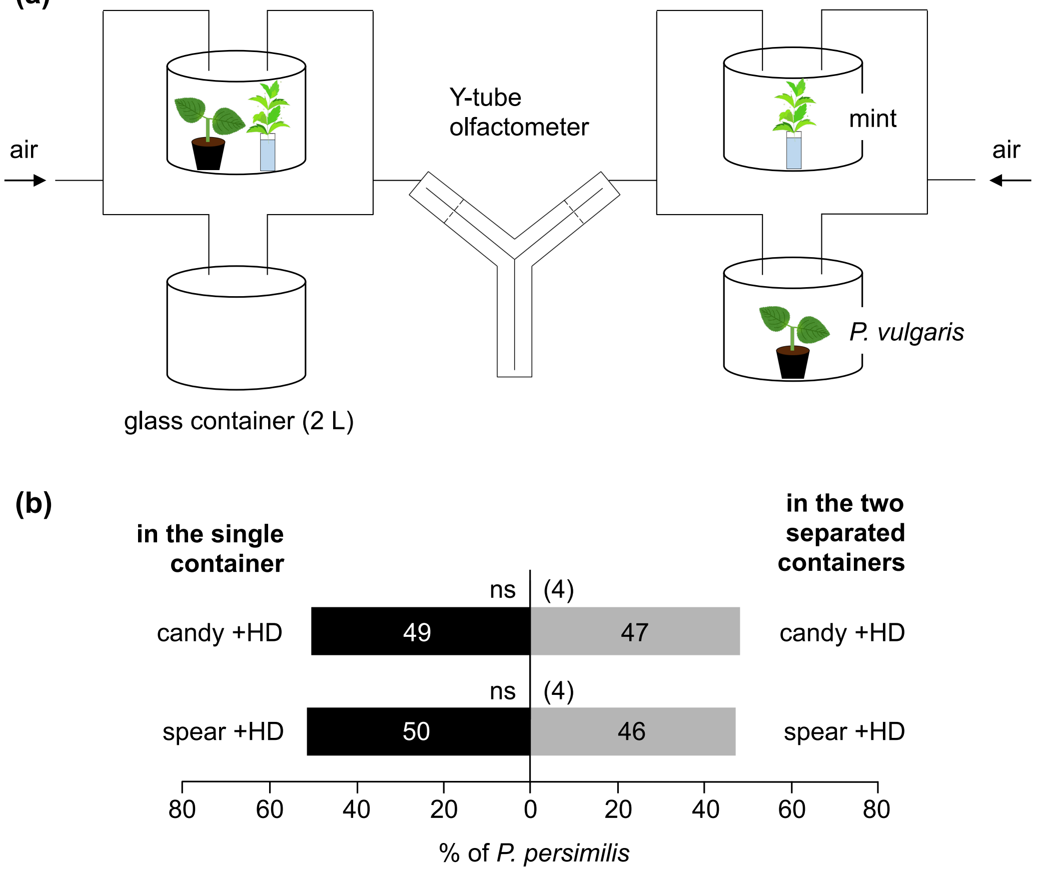

Supplemental Fig.2

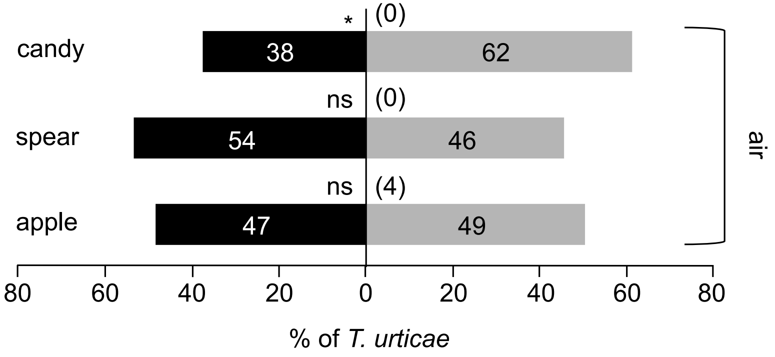

Supplemental Fig.3

Supplemental Figure 1. Olfactory response of *Phytoseiulus persimilis* to individual mint VOCs of candy mint and spearmint, and to their respective blends. The numbers in the bars indicate the numbers of predatory mite females that made choices. The figures in parentheses represent the numbers of predators that did not choose either odor source (‘no choice’ subjects). A replicated G-test was conducted to evaluate the significance of attraction in each experiment (ns, $P > 0.05$).

Supplemental Figure 2. Olfactory response of *Phytoseiulus persimilis* when offered potted, heavily damaged (HD) *Phaseolus vulgaris* plant enclosed with candy mint or spearmint plantlets (4 g fresh weight) together in a single container vs. the potted HD plant enclosed separately with candy mint or spearmint plantlets in two separated containers in a Y-tube olfactometer. The experimental design of this assay is shown in (a). The data are shown in (b). The numbers in the bars indicate the numbers of predatory mite females that made choices. The figures in parentheses represent the numbers of predators that did not choose either odor source (‘no choice’ subjects). A replicated G-test was conducted to evaluate no significance of attraction in each experiment (ns, $P > 0.05$).

Supplemental Figure 3. Olfactory response of *Tetranychus urticae* when offered candy mint, spearmint or apple mint plantlets (4 g fresh weight) vs. clean air in a Y-tube olfactometer. The numbers in the bars indicate the numbers of *T. urticae* females that made choices. The figures in parentheses represent the numbers of mites that did not choose either odor source (‘no choice’ subjects). A replicated G-test was conducted to evaluate the significance of attraction in each experiment (*$P < 0.05$; ns, $P > 0.05$).
